# Life without Roe v Wade

**DOI:** 10.1186/s40834-021-00149-6

**Published:** 2021-03-01

**Authors:** Norman A. Ginsberg, Lee P. Shulman

**Affiliations:** 1 Prentice Hospital Northwestern University Feinberg School of Medicine, 250 E Superior St, Chicago, IL 60611 USA; 2 Highland Park, USA

*Roe v Wade* (RVW) was a landmark legal decision issued on January 22, 1973. With this decision, the US Supreme Court stuck down a Texas statute banning abortion. Prior to this decision, abortion had been illegal through out most of the country since the late nineteenth century. This decision set forward a legal precedent affecting 30 subsequent Supreme Court cases regarding abortion. However, the Supreme Court is now predominately conservative and is potentially poised to reverse this decision. NARAL, a pro-choice American organization, estimates that if RVW is overturned that abortion will become illegal in 17 states [1].

RVW was announced 47 years ago, meaning that no current woman of reproductive age (15–44) has any personal knowledge of how things were before the decision was pronounced. The average age of physicians in the US today is 51; therefore, most physicians also do not have any personal knowledge of the clinical issues before RVW.

The purpose of this article is to reacquaint women and physicians as what to expect if or when abortion becomes illegal, as the saying goes “… those that forget history are doomed to repeat it.” This is not written to favor one moral philosophy over another and is not meant to be a political tome. Rather, it is penned to provide information and experiences that may be needed in the future.

I remember vividly my medical student rotation at Cook County Hospital before RVW. Within the hospital there was a 40-bed ward called ward 41, usually filled to capacity with women that had suffered complications of illegal abortions. Those who obtained illegal abortions had them performed under a great veil of secrecy. They were often blindfolded and whisked off to some unknown place. Practitioners of this method did what ever they could to avoid police detection. The poor and those from minority groups who couldn’t access abortion safely turned to a variety of methods, including: self-medication with toxic chemicals such as turpentine, bleach, detergent solutions quinine, and strong teas. Other women used a vaginal approach with potassium permanganate tablets and herbal preparations. Foreign bodies were commonly placed into the uterus through the cervix and commonly included sticks dipped in oil, wire, knitting needles, coat hangers, ball point pens and air blown in by either a syringe or turkey baster. The use of air could lead to air embolism and death. In addition, women would frequently resort to enemas and vaginal or abdominal trauma.

A section near the emergency room was set aside for triaging these women. Chemical burns and perforations of the vagina, bladder, uterus and rectum were frequently detected. Many of these women came in with overwhelming infections, septic shock or heavy bleeding. The role of triage was to determine which women needed immediate surgery or those who required medical interventions. Death in the ward, which was just a series of beds separated by a curtain, was a common occurrence at a rate of more than one per month. It should be noted that today we have superior and stronger antibiotics than in the 1970s; however, the decades of use of these antibiotics have led to the development of resistant strains that have reduced the effectiveness of these and subsequent therapeutics.

This recounting portrays what occurred, in most ways, across the nation. Women then, like today, had similar reasons for ending their pregnancies such as having a baby would drastically interfere with their education, work, care for other dependents or they just couldn’t afford a baby, in addition to accused rape and incest. Many of them would plan to have a child when the circumstances were right; unfortunately, a great number of those lost the ability to become pregnant again. When abortion became legal that ward disappeared and with it the enormous number of medical and surgical complications, as well the emotional trauma associated with illegal abortion and its complications [2, 3].

Abortion is one of the oldest events in recorded history; the procedure can be traced back as far as 2700 BCE. Abortion was first practiced in China, and then in ancient Egypt. Artistic renderings of the process can be found on a series of friezes as a bas-relief at Angkor Wat in Cambodia [4].. Without question, abortion is a fundamental part of human history that is not a new procedure and is not likely to “go away” by making it illegal.

The CDC estimates that 14% of all pregnancies are unwanted [5]. Today the incidence of securing an abortion in countries that restrict abortion and those with more liberal access are surprisingly equivalent [6–9]. That fact highlights the futility of make abortion illegal since it still continues unabatedly. Successful use of contraception could result in 4.5 million fewer abortions worldwide [9]. Unfortunately, no method is 100% effective in preventing pregnancy, though some methods (e.g., LARC methods) are more effective than others (e.g., oral contraceptives). Indeed, all methods carry some risk for failure, including permanent sterilization procedures (e.g., tubal ligation, vasectomy). For those that wish to overturn RVW, greater effort needs to be exercised in making contraception widely available.

United States data suggest that legal abortion has no more risk than other minor surgical procedures. These data clearly support the safety of legal abortion in comparison to the risks associated with pregnancy. Indeed, pregnancy is associated with a 14-times greater maternal mortality rate in comparison to abortion. Social stigma often prevents women from seeking timely help after an illegal abortion, which in turn may lead to severe disability or death [10]. Worldwide, an estimate of 68,000 women (eight/hour) die as a result of an illegal abortion. This translates to a case-fatality rate of 367/100,000, which in turn is hundreds of times greater than legal abortions. The World health Organization reported that globally the case fatality rate is 350 times that of legal abortions [7, 8, 11]. It can be expected that the maternal death rate will increase in the absence of legal abortions [6].

If the Supreme Court were to reverse RVW, there could be serious consequences for those who choose abortion to deal with their unwanted pregnancy. Recently, Georgia passed House Bill 481, which conferred “natural person” to an embryo with a heartbeat at 6 weeks of gestation. A person would then be committing murder by causing the death of another human being (natural person). In that scenario people that had anything to do with the termination, including the mother, doctor, nurse or pharmacist, could be caught up in the legal proceeding and potentially be charged as accessories to murder. At this point it is still unclear if this will survive a legal challenge or if a prosecutor would actually try a case of murder under these circumstances.

Many women unable to get an abortion will bring their pregnancy to term. Numerous women have told me that pregnancy was the best time of their life. Will it be possible for women, in this situation, to be able to enjoy their pregnancy? Will the moving and kicking fetus be a delight or be a harbinger of what they will lose? The nine months of pregnancy will undeniably create some bonding of the mother to the fetus. Labor will approach, a difficult, long and arduous process. After the delivery they will have the baby for a few days until the adoption can be arranged. Relinquishing the baby will be an emotionally wrenching experience. There will be mixed emotions along with feelings of failure and loss in addition to some relief that the ordeal had ended. Every year, they will remember the child they gave up, wondering about what they are missing and if they made the right choice. And they will never forget.

The next consideration is for neonates that are born with congenital malformations. In a study performed in Sweden it was reported that congenital anomalies occurred in 7.6% of newborns. Approximately 46% of the detected anomalies were minor but 55% were classified as severe. One consequence of banning abortion will be the increase in severely handicapped children. Caring for a child with physical or emotional disabilities is enormously expensive from both a financial and emotional consideration. Where will this money come from? As of now the financial burden mostly falls on the parents [12]. Schecter KB et al. reported a study conducted over 13 years where they examined 53,000 pregnancies. Anomalies were graded into 4 groups: in group 1 there was no impact on quality of life, in group 2 little impact on quality of life, group 3 this category was serious impacting on quality of life, even with optimal medical therapy and group 4 was not compatible with life. For the mildest abnormality the abortion rate was 0.9% and for group 3 it rose to 72.5%. Maternal age directly correlates with management of group 3. The older the mother, the more likely she will end the pregnancy. Serious congenital anomalies may disproportionately affect children with the youngest mothers, since they were the most likely to continue the pregnancy [8]. Last year in the United States, 3,788,235 children were born. Of those born approximately 6% (227,294) will have an anomaly. It can be expect that at least 50% of those would be severe (113,647) in the group 3 [13].

For those who do not avail themselves with abortion, there is apprehension that the rate of child maltreatment is likely to increase when families are faced with an unintended pregnancy. Guterman [5], reported a study looking at the prospective of both parents faced with an unplanned pregnancy. Drawing upon data, from the Fragile Families and Child Well Being Study, a survey was taken after birth regarding whether the couple had considered abortion. Whether the mother or father viewed it as an unintended pregnancy, the relationship with maltreatment behavior was largely the same for both parents. The mothers expressed the maltreatment in the form of psychological aggression and neglect. The fathers expressed maltreatment more in the form of physical aggression [14].

How will low-income families deal with the additional burden of another child in the family when they are already living from paycheck to paycheck? Two years ago, about 75% of US workers said they were living from payday to payday, a number that has grown to 78% in 2019 and has likely increased even more with the worldwide adverse economic impact of the COVID-19 pandemic. The study conducted by Harris Poll, surveyed nearly 2400 hiring and human resource-managers and 3500 adult employees who worked full-time in June and May to derive these figures.

What will become of the unwanted children? Will we again see an increase in orphanages and foster care? Short of wars and natural disasters, poverty along with drugs and alcohol are leading causes for abandonment. Abandoned child syndrome is a proposed behavioral or psychological condition that results primarily from the loss of one or more parents. Abandonment can be considered physical when the parents are out of the child’s life or emotional when the parents withhold affection, nurturing, or stimulation. At the present time there are roughly 400,000 children in US foster care. I can remember growing up and seeing numerous orphanages’ when abortion was illegal. It will be heartbreaking see more and more children abandoned because they were unwanted. Additionally there were homes for unwed mothers that had no other types of support. Might that also come back?

Reflecting on the up side, there will be more children available for adoption if abortion is unobtainable. Couples that have failed to conceive despite treatment with state-of-the-art fertility treatments centers will be able to adopt a child without needing to resort to go to China or Russia. However, it is important to recognize that there is still an overabundance of children waiting to be adopted. These children spend an average of 3 years in foster care before they are adopted. Accordingly, an increase in the number of children available for adoption may profoundly tax a system that strives to place children with caring and supportive parents.

Undoubtedly, life for many would be more problematic in the absence of RVW. These facts explain why so many groups of women fought so hard to maintain RVW. On the other side, we can expect there to be more liveborn children. Many of these children will be a blessing in the lives of their family. Some may go on to be great scholars, lawyers and politicians in turn greatly contributing to society. Some examples of orphaned children are Steve Jobs, Ray Charles, Marilyn Monroe, Eleanor Roosevelt, Babe Ruth and Herbert Hoover. However, many of these children may be abandoned or abused by parents that did not want them in the first place. Adding to that the surgical and medical trauma that is likely to accrue to an increasing number of women.

During 2020 we were woefully unprepared for the viral pandemic, in part by a lack of knowledge on how to best manage it. It is hope that this small message of a time long ago will help future generations to be better prepared if RVW is reversed and we are forced to return to that time of frequent illicit abortion.

## Data Availability

This data was obtained from the literature referenced in the manuscript and publically available.
